# Alterations in Gene Expression of Proprotein Convertases in Human Lung Cancer Have a Limited Number of Scenarios

**DOI:** 10.1371/journal.pone.0055752

**Published:** 2013-02-07

**Authors:** Ilya V. Demidyuk, Andrey V. Shubin, Eugene V. Gasanov, Alexander M. Kurinov, Vladimir V. Demkin, Tatyana V. Vinogradova, Marina V. Zinovyeva, Alexander V. Sass, Irina B. Zborovskaya, Sergey V. Kostrov

**Affiliations:** 1 Institute of Molecular Genetics, Russian Academy of Sciences, Moscow, Russia; 2 Shemyakin and Ovchinnikov Institute of Bioorganic Chemistry, Russian Academy of Sciences, Moscow, Russia; 3 Blokhin Cancer Research Center, Russian Academy of Medical Sciences, Moscow, Russia; Ospedale Pediatrico Bambino Gesu', Italy

## Abstract

Proprotein convertases (PCs) is a protein family which includes nine highly specific subtilisin-like serine endopeptidases in mammals. The system of PCs is involved in carcinogenesis and levels of PC mRNAs alter in cancer, which suggests expression status of PCs as a possible marker for cancer typing and prognosis. The goal of this work was to assess the information value of expression profiling of PC genes. Quantitative polymerase chain reaction was used for the first time to analyze mRNA levels of all PC genes as well as matrix metalloproteinase genes *MMP2* and *MMP14*, which are substrates of PCs, in 30 matched pairs of samples of human lung cancer tumor and adjacent tissues without pathology. Significant changes in the expression of PCs have been revealed in tumor tissues: increased *FURIN* mRNA level (p<0.00005) and decreased mRNA levels of *PCSK2* (p<0.007), *PCSK5* (p<0.0002), *PCSK7* (p<0.002), *PCSK9* (p<0.00008), and *MBTPS1* (p<0.00004) as well as a tendency to increase in the level of *PCSK1* mRNA. Four distinct groups of samples have been identified by cluster analysis of the expression patterns of PC genes in tumor vs. normal tissue. Three of these groups covering 80% of samples feature a strong elevation in the expression of a single gene in cancer: *FURIN*, *PCSK1*, or *PCSK6*. Thus, the changes in the expression of PC genes have a limited number of scenarios, which may reflect different pathways of tumor development and cryptic features of tumors. This finding allows to consider the mRNAs of PC genes as potentially important tumor markers.

## Introduction

Proprotein convertases (PCs) is a protein family which includes nine highly specific subtilisin-like serine endopeptidases in mammals (reviewed in [1]). The key function of these enzymes is processing and/or activation of numerous proteins and peptides. Endogenous substrates of PCs include neuropeptides, peptide hormones, growth and differentiation factors, adhesion molecules, extracellular matrix proteins, receptors, enzymes, blood coagulation factors, and plasma proteins. In addition, pathogenic viruses and bacteria can use host PCs to switch on their proteins such as viral coat proteins or bacterial toxins. Since the activation of proproteins in the right time and place is clearly crucial for homeostasis, PCs are involved in the control of various physiological processes in health and disease. PCs as a processing system feature a combination of specificity and redundancy [2]: each protein of the group has unique structural and functional properties; at the same time, the properties of PCs overlap. The specificity and redundancy are observed not only at the levels of substrate specificity and cellular localization but also for the temporal/tissue profiles and, possibly, for the regulation mechanisms of gene expression. In this context, the identification of individual physiological properties and natural partners of enzymes of this group is not an easy matter, which can be properly solved only when PCs are considered as an integrated system.

Many substrates of PCs are associated with malignant diseases. For instance, the direct involvement in tumor progression and metastasis has been demonstrated for insulin-like growth factor 1 (IGF-1) and its receptor (IGF-1R), transforming growth factor β (TGF-β), vascular endothelial growth factor C (VEGF-C), and matrix metalloproteinases (MMPs) (reviewed in [3]). By activating the key cancer-associated proteins, PCs have an effect on cell proliferation, motility, and adhesion as well as tumor invasion, which suggest PCs as promising therapeutic targets [4].

The first data on the association of PCs with cancer were published in 1987 [5]. Since then, numerous studies analyzed the expression of PCs in cancer and the correlations between the PC expression levels and cancer properties using various experimental approaches [6]–[21]. Overall, the data obtained demonstrated altered levels of PC mRNAs in cancer. Correlations between PC expression profiles and cancer aggressiveness [9], [12], [16], survival rate [18], and neuroendocrine differentiation of cancer cells [6], [7], [13] were shown. This allows us to propose the expression status of the PC system as a possible marker for cancer typing and prognosis.

Lung cancer is the most widespread oncological disease, which causes 1.4 million deaths annually [22]. Not surprisingly, PC expression data were first obtained for this cancer type [5]–[7]. These as well as more recent publications [8], [11]–[13] demonstrated altered expression of *FURIN*, *PCSK1*, *PCSK2*, and *PCSK6* genes in lung cancer. (Hereafter, gene symbols follow the recommendations of the HUGO Gene Nomenclature Committee, www.genenames.org. The corresponding common protein designations are given in Table 1.) High *FURIN* expression was found in non-small cell lung carcinomas (NSCLCs) vs. small cell lung carcinomas (SCLCs) [5], [8] and correlated with the aggressiveness of lung cancer cell lines [12]. *PCSK1* and *PCSK2* expression is largely detected in cancers with neuroendocrine features, in particular, SCLC [6]–[8], [11], [13]. At the same time, *PCSK6* expression is not necessarily observed in lung cancer, although it is more common in NSCLC than in SCLC [8]. Thus, the response of the PC system varies with lung cancer types. Gene expression surveys involving microarray analysis (including whole-transcriptome ones) demonstrate a substantial heterogeneity of lung cancer samples [13], [23]–[26], and the revealed differences correlate with the patients' survival rate [23], [24], [26]. Overall, this proposes lung cancer as a test system to assess the information value of the approach based on expression profiling of PC genes.

**Table 1:** Gene designations and TaqMan Gene Expression Assays used in real-time PCR.

Gene/protein name	Common protein designation	HGHC gene symbol*	Assay ID
Furin	Furin	*FURIN*	Hs00159829_m1
Membrane-bound transcription factor peptidase, site 1	SKI-1/S1P	*MBTPS1*	Hs00921626_m1
Proprotein convertase subtilisin/kexin type 1	PC1/3	*PCSK1*	Hs00175619_m1
Proprotein convertase subtilisin/kexin type 2	PC2	*PCSK2*	Hs00159922_m1
Proprotein convertase subtilisin/kexin type 4	PC4	*PCSK4*	Hs00399493_m1
Proprotein convertase subtilisin/kexin type 5	PC5/6	*PCSK5*	Hs00196400_m1
Proprotein convertase subtilisin/kexin type 6	PACE4	*PCSK6*	Hs00159844_m1
Proprotein convertase subtilisin/kexin type 7	PC7	*PCSK7*	Hs00237114_m1
Proprotein convertase subtilisin/kexin type 9	NARC-1/PCSK9	*PCSK9*	Hs00545399_m1
Matrix metallopeptidase 2	MMP2	*MMP2*	Hs00234422_m1
Matrix metallopeptidase 14	MT1-MMP	*MMP14*	Hs00237119_m1

* HGNC – HUGO Gene Nomenclature Committee (www.genenames.org).

In this context, the present work for the first time evaluated mRNA levels of all PC genes in lung cancer using reverse transcription followed by a quantitative real-time polymerase chain reaction (qPCR). In addition, we studied the gene expression of two matrix metalloproteinases (MMP2 and MMP14), which are key factors in cancer invasion and metastasis [27] and substrates of PCs [28]–[30].

## Materials and Methods

### Ethics Statement

The research was approved by the Institutional Review Board of Blokhin Cancer Research Center (Moscow, Russia), and written informed consent was obtained from each patient involved in the study.

### Collection of tissue samples

**Figure 1:**
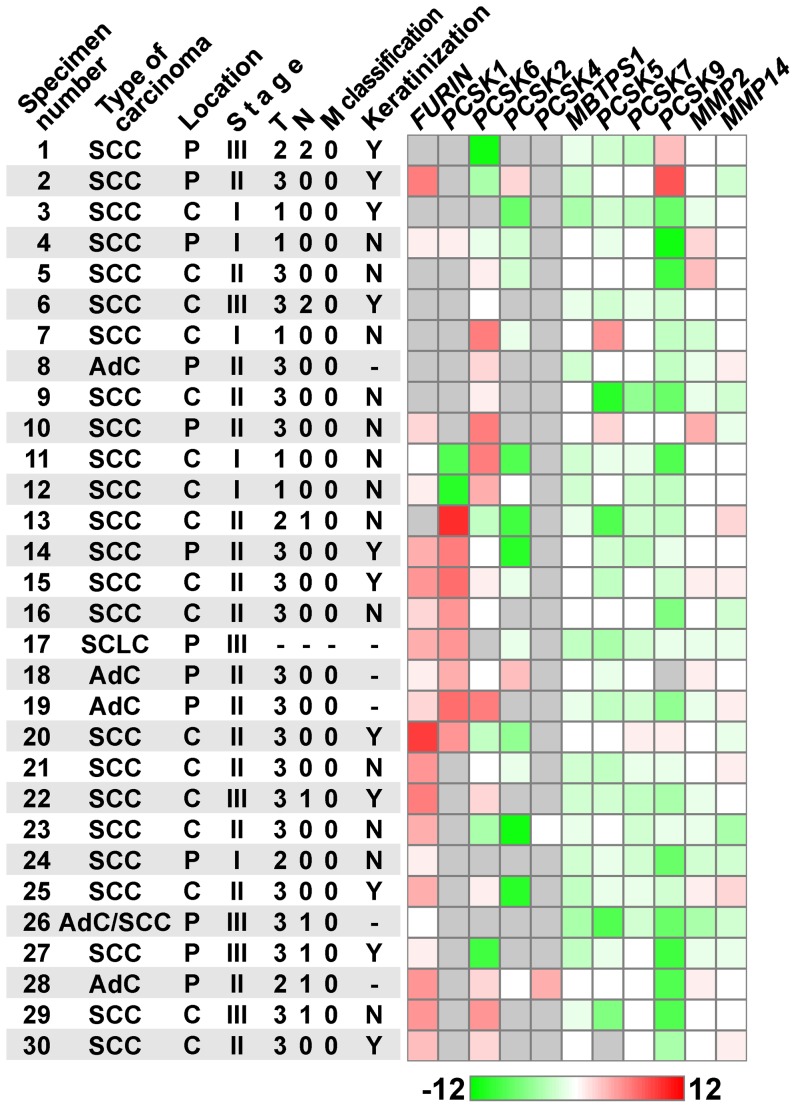
Description of the specimens studied and heat map presentation of the ratio values of gene expression in tumor vs. adjacent tissues without histological pathology. SCC, squamous cell lung carcinoma; AdC, adenocarcinoma; AdC/SCC, both AdC and SCC cells were found in the tumor tissue; SCLC, small cell lung carcinoma; P, peripheral tumor location; C, central tumor location; Y, tumor with keratinization; N, tumor without keratinization; ‘-’, no data. The heat map is shown in log_2_ scale. Gray cells indicate specimens with undetectable mRNA in both tumor and normal tissues.

Specimens of cancer tumor tissues and of adjacent tissues without histological pathology (further referred to as normal tissue) were taken from 30 patients with diagnosed small cell lung carcinoma and non-small cell lung carcinoma (tumor stage I–III) during surgery (Figure 1, Table S1). In every case localization of a primary tumor node was determined. If a tumor originated from the smallest bronchi in peripheral segments of lung and had no connection with bronchi lumen, its localization was considered peripheral. If a tumor originated from a large bronchus, its localization was considered central. The normal tissue specimens were taken from the edge of resections (the distance between tumor and normal tissues was no less than 20 mm). All patients were under medical supervision in the Blokhin Cancer Research Center (Moscow, Russia) during a period from May 2004 to November 2005. None of these patients received radio or chemical therapy to the moment of the investigation.

Each specimen was split into two portions. The first one was immediately frozen in liquid nitrogen for mRNA isolation. The second portion was used for histological examination after hematoxylin and eosin staining of the paraffin sections. Tumor tissue specimens contained more than 70% of malignant cells. In normal tissue specimens, no malignant cells were found. For SCC samples presence of keratinazation was determined. Existence of keratinization allowed to refer a sample to a group of well-differentiated cancer.

### RNA isolation and purification

The total RNA was isolated from homogenized tumor or normal tissues by guanidine isothiocyanate lysis and acid-phenol extraction with subsequent removal of polysaccharide admixtures [31]. Additional purification was performed by RNA precipitation using an RNeasy Mini kit (Qiagen, USA). Further treatment with DNase I (Promega, USA) was done according to the supplier's recommendations. The obtained RNA samples were characterized electrophoretically in 1% agarose gel. RNA concentration was determined by spectrophotometry.

### Double-stranded cDNA synthesis

Oligonucleotides AAGCAGTGGTATCAACGCAGAGTACGCrGrGrG and AAGCAGTGGTATCAACGCAGAGTACT_30_VN (V = C, or G, or A) (Syntol, Russia) were used in the reverse transcription reaction. For the first strand cDNA synthesis, 1 µg of isolated RNA was incubated with reverse transcriptase PowerScript (Clontech, USA) as described by Y. Zhu et al. [32]. The obtained reaction mixture was used for the second strand synthesis followed by PCR using the Advantage 2 DNA polymerase (Clontech, USA) and primer AAGCAGTGGTATCAACGCAGAGT under the following conditions: 95°C for 1.5 min; up to 17 cycles of 95°C for 20 s; 65°C for 20 s; and 72°C for 3 min. To obtain equal amounts of all amplification products, the number of cycles varied (commonly, 15 cycles).

### Real-time PCR

Real-time PCR was performed using the primers and probes of the TaqMan Gene Expression Assays system (Applied Biosystems, USA) (Table 1). TaqMan Pre-Developed Assay Reagent GAPDH 20× (Applied Biosystems, USA) was used to quantify the reference gene, glyceraldehyde 3-phosphate dehydrogenase (*GAPDH*). PCR was conducted using a Chromo4 Dyad Disciple cycler (BioRad, USA) according to the supplier's recommendations with the following program: 50°C for 2 min; 95°C for 10 min; 45 cycles of 95°C for 15 s and 60°C for 60 s; the reaction volume was 20 µl. Every sample was tested at least twice in duplicates. The threshold cycle was defined using the Opticon Monitor 3 software (BioRad, USA).

### Experimental data processing

The experimental data obtained for genes under study were normalized to *GAPDH* mRNA levels using the formula: 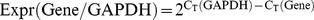, and the results were averaged (Table S1). The values for tumor and normal tissues were designated as Expr^T^ and Expr^N^, respectively. The Expr^T^ to Expr^N^ ratio (Ratio^T/N^) and 95% confidence intervals were calculated for each gene.

In some samples, mRNAs of certain genes were not detected in tumor or normal tissues in one of two independent experiments. In these cases, the Expr and Ratio values were calculated from the data of the other experiment. In some samples, real-time PCR failed to detect mRNAs of certain genes in tumor or normal tissues in both experiments; in these cases the C_T_ was set equal to 42 in the Ratio^T/N^ calculations. If mRNAs were undetectable in tumor and normal tissues in both experiments, the Ratio^T/N^ values were not calculated.

### Statistical analyses

Wilcoxon matched-pairs rank-sum test was used to evaluate the significance of difference between the mRNA levels of genes in tumor and normal tissues. Kruskal-Wallis one-way analysis of variance was performed to evaluate the influence of tumor type, stage, and TNM characteristics on mRNA levels of the studied genes. Spearman rank correlation coefficients were calculated to evaluate the relationship between pairs of gene expression profiles. Cluster analyses of gene expression patterns and expression profiles were performed for the Expr and Ratio^T/N^ values by the Ward method using Spearman rank correlation coefficients as the distance measure. All above statistical analyses were performed using the Statistica 8.0 software (StatSoft, USA). Heat maps were built using the Matrix2png software tool [33].

## Results and Discussion

In this work, quantitative PCR was used for the first time to analyze mRNA levels of all PC genes (listed in Table 1) as well as matrix metalloproteinase genes *MMP2* and *MMP14* in 30 matched pairs of samples of human lung cancer tumor and adjacent normal tissues (Table S1, Figure 1). Expression of *MBTPS1*, *PCSK7*, *MMP2*, and *MMP14* was observed in all tumor and normal tissue samples; and *PCSK5*, in almost all samples. Conversely, *PCSK4* mRNA was detected in two tumor samples only. The expression profiles of other genes were more complex. *PCSK9* transcript was found in 29 normal tissue samples but only in 18 tumor ones. *PCSK6* expression was detected in about two thirds of normal and tumor tissues; and *PCSK2*, in 15 and 11, respectively. The most pronounced differences between normal and tumor tissue expression was observed for *FURIN* and *PCSK1*. The samples where mRNAs of these genes were detected were twice more frequent in tumor than in normal tissues, while their expression was undetectable in a substantial fraction of both tumor (21/30 for *PCSK1* and 8/30 for *FURIN*) and normal samples (26/30 and 20/30, respectively). Overall, these findings demonstrate significant differences in the expression of individual PC genes in the human lung, on the one hand, and high variation in their expression patterns (i.e., combinations of expression levels of the genes) between individuals, on the other hand.

The data obtained are, in general, in good agreement with published results. *PCSK4* was shown to be largely limited to testicular and ovarian germ cells [34]–[36]. PCSK1 and PCSK2, the principal activators of prohormones and proneuropeptides within the regulated secretory pathway, are largely detected in neural and endocrine cells [37]. All other genes studied (encoding both PCs and MMPs) are commonly reported as ubiquitously or widely expressed. As well as other authors, we found mRNA of *MBTPS1*, *PCSK5*, *MMP2*, and *MMP14* in all or practically all the samples of tumor and normal lung tissues (e.g. [38], [39], datasets GDS1650, GDS1673, and GDS2491 in the Gene Expression Omnibus database at www.ncbi.nlm.nih.gov/geo/). In case of PCSK6, in full conformity to other published data ([8], GDS1650, GDS1673, and GDS2491), we found the mRNA not in all, but major portion of the samples analyzed. Our results concerning *PCSK9* are also in agreement with the published data (GDS1673), although the current information about expression of this gene in lung is scarce. We found *FURIN* mRNA in approximately half of all samples, which is in agreement with data obtained by microarray technology (GDS1650, GDS1673, and GDS2491), but in contradiction to published data acquired by Nothern blot analysis [5], [8]. The reason of this discrepancy may be explained by features of the methods used. The largest inconsistency concerns *PCSK7*. There are few data about its expression in lung. The data presented so far are obtained with microarray technology and do not match to each other. We found *PCSK7* mRNA in all our samples, Gruber with coworkers found it in 14 out of 40 of not-diseased lung samples ([40] and GDS1673), and Stearman with colleagues did not find it in any of 20 tumor and 19 normal lung tissue samples ([41] and GDS1650). It does not seem possible to explain reasons of the distinctions, but it seems most probably to be due to differences in the experimental platforms used: qPCR and various generations of Affymetrix chips.

Thus, the obtained and published data demonstrate a high variation in PC gene expression between individuals. This gives no grounds to expect that mRNA levels of PCs in tumor or normal tissue alone can have any prognostic value or can be used for cancer typing. Indeed, no significant differences between the expression levels of the genes analyzed or their expression patterns have been revealed for groups of normal or cancer samples with similar clinical features. Likewise, cluster analysis failed to reveal groups of samples with similar gene expression patterns.

At the same time, we have found moderate but significant (p<0.05) pairwise correlations between the expression profiles of the genes studied (Table 2). Note that the sets of correlated profiles substantially differed for tumor and normal tissues. Assuming that the revealed correlations indicate the coordinated regulation of gene expression, one can propose that the regulation of expression of PCs and MMPs is modified in lung cancer. It is important to note, this applies to the great majority of PC genes. Moreover, the correlations between the profiles of changes in the expression in tumor vs. normal tissues (differential expression profiles) can be attributed to the mechanisms underlying the expression changes common for several genes.

**Table 2:** Correlations between expression profiles of the genes studied.

*Normal tissue*								
Gene pairs	*FURIN PCSK5*	*FURIN MMP14*	*MBTPS1 PCSK7*	*MBTPS1 PCSK5*	*MBTPS1 MMP2*	*PCSK7 PCSK5*	*PCSK9 PCSK2*	*PCSK9 PCSK5*
**R_s_** *	0.64	−0.67	0.53	0.45	0.65	0.38	0.78	0.58

*Tumor tissue*							
Gene pairs	*FURIN PCSK7*	*FURIN MBTPS1*	*MBTPS1 PCSK6*	*MBTPS1 PCSK7*	*MBTPS1 MMP2*	*PCSK6 MMP2*	*PCSK7 MMP2*
**R_s_** *	0.56	0.53	0.63	0.42	0.65	0.47	0.51

*Tumor tissue/Normal tissue*							
Gene pairs	*FURIN PCSK1*	*FURIN PCSK9*	*MBTPS1 PCSK7*	*MBTPS1 MMP2*	*PCSK1 PCSK5*	*PCSK7 PCSK5*	*PCSK7 MMP2*
**R_s_** *	0.77	0.51	0.64	0.65	−0.66	0.61	0.61

* R_s_ is Spearman correlation coefficient (p<0.05).

Analysis of mRNA levels of the studied genes demonstrated significant differences between tumor and normal tissues: the average level of *FURIN* mRNA increased (p<0.00005); mRNA levels of *PCSK2* (p<0.007), *PCSK5* (p<0.0002), *PCSK7* (p<0.002), *PCSK9* (p<0.00008), and *MBTPS1* (p<0.00004) decreased; while *PCSK1* mRNA level showed a tendency to increase (Figure 1). Thus, the expression of seven out of eight PC genes (except *PCSK6*), whose mRNA is detectable in the lung, demonstrated unidirectional changes in lung cancer in our samples. Although the expression of PCs was analyzed in many publications, this is an original finding, since mRNA levels of most PC genes in tumor vs. normal tissues have not been quantified previously. At the same time, the high level of *FURIN* expression in NSCLC [5], [8] and other cancer types [10], [16], [18] has been reported previously.

The role of MMPs in cancer progression as regulators of the tumor microenvironment is currently receiving much attention (reviewed in [42], [43]). In this study we analyzed expression of two MMP genes of different types: secreted MMP2 и membrane-anchored MMP14. These proteases are the major MMPs involved in cancer cell invasion and proliferation, tumor angiogenesis and vasculogenesis, cell adhesion and migration as well as in immune surveillance. Taking this into account, the absence of significant differences between *MMP2* and *MMP14* expression levels in tumor and adjacent tissues without histological pathology can look surprising. However, this result is in agreement with ample evidence for high levels of their expression both in cancer and stromal cells in NSCLC [38], [39], [44]–[52]. At the same time, a direct comparison of *MMP2* and *MMP14* expression in cancer tumor vs. adjacent normal tissues was reported only in two publications, and their conclusions are at variance. The former, similar to our study, observed significant differences for neither *MMP2* nor *MMP14* [46]. The latter publication demonstrated an elevated expression of *MMP14* in cancer relative to normal lung specimens [39]. Most likely, this discrepancy is due to different specimen types analyzed: squamous cell carcinomas (SCCs) prevailed in the former study [46] and in our work, while more adenocarcinomas (AdCs) were analyzed in the latter report [39].

**Figure 2:**
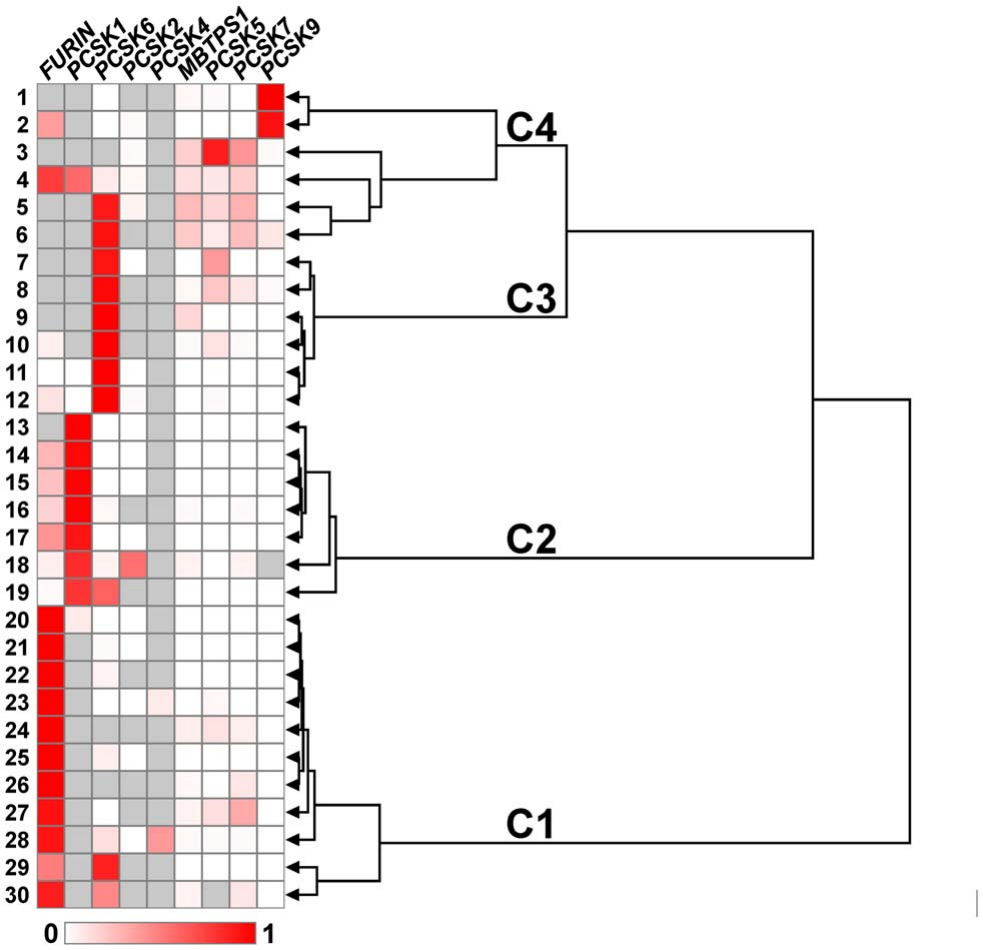
Clustering of PC genes' expression data. Specimens numbering corresponds to Fig. 1. The Ratio^T/N^ values in the heat map were row-normalized and shown in linear scale. Gray cells indicate specimens with undetectable mRNA in both normal and cancer tissues. Branch length reflects the distance between the dendrogram nodes. The clusters found are marked as C1, C2, C3 and C4.

In our view, the most prominent result was obtained by comparing the patterns of changes in the expression of PC genes between tumor and normal tissues. Cluster analysis divided studied samples into four groups (Figure 2), which did not correlate with the available clinical features of tumors. Three of these groups (C1, C2, and C3) cover 80% of samples. Each group is rather homogeneous and has a single key gene: *FURIN* in C1, *PCSK1* in C2, and *PCSK6* in C3. Usually, the key gene expression is elevated in cancer; although, it can be unaltered or slightly decreased against the background of a substantial decrease in mRNA levels of other PCs (Figure 1). Undetectable *PCSK6* expression in most samples is an extra character of C1, while C3 features undetectable expression of *PCSK1* and/or *FURIN* in more than a half of samples. C4 is more heterogeneous. The samples in this group share similar expression changes of *PCSK5*, *PCSK7*, *PCSK9*, and *MBTPS1* as well as undetectable mRNAs of *FURIN* and *PCSK1* in most cases. Thus, the changes in the expression of PC genes in lung cancer have a limited number of scenarios, which may correspond to previously undetected NSCLC types.

It is of interest that the enzymes encoded by the key genes of the revealed groups belong to different types of PCs [53]. Furin, PCSK1, and PCSK6 have different kinds of C-terminal extensions. These PCs have different expression profiles: PCSK1 is localized in neural and endocrine cells, PCSK6 occurs widely, and furin is ubiquitous. Finally, they exhibit different secretion patterns: PCSK1 follows the regulated secretory pathway, while furin and PCSK6 are constitutively secreted. In this context, one can propose that different scenarios of alteration in the expression of PC genes induce different changes in the range of activated substrates.

The data available to date can provide only hints about the origin of the revealed groups. For instance, C2 with active *PCSK1* can correspond to NSCLCs with signs of neuroendocrine differentiation [54]–[65], which can point to the origin of these tumors. The formation of C1 (*FURIN*) and C3 (*PCSK6*) can be mediated by the E2F1 transcription factor, which specifically upregulates *PCSK6* but not *FURIN* or *PCSK5* [66]. However, these data provide no reliable explanation of the mechanisms underlying the typical scenarios of changes in the transcription of PCs in lung cancer. Still, at least two radically different considerations can be advanced. On the one hand, the observed effects can stem from the differences that existed before malignant transformation, e.g., genotype differences between individuals or cell type differences within an individual. On the other hand, it can be due to the alterations emerged during cancer formation, in particular, local disorders in the expression of individual PC genes or varieties of the global dysregulation of gene expression. Moreover, the observed events can result from the effect of several factors at the same time.

Overall, analysis of the patterns of changes in the expression of PC genes in individuals allowed us to reveal several NSCLC types and to demonstrate that the expression changes have a limited number of scenarios, which may reflect different pathways of tumor development and cryptic features of tumors. This finding warrants further investigation, and allows to consider the mRNAs of PC genes as potentially important tumor markers.

## Supporting Information

Table S1 **Characteristics of specimens and gene expression data.**
(XLS)
